# Gut bacterial communities of diarrheic patients with indications of *Clostridioides difficile* infection

**DOI:** 10.1038/sdata.2017.152

**Published:** 2017-10-17

**Authors:** Dominik Schneider, Andrea Thürmer, Kathleen Gollnow, Raimond Lugert, Katrin Gunka, Uwe Groß, Rolf Daniel

**Affiliations:** 1 Genomic and Applied Microbiology and Göttingen Genomics Laboratory, Institute of Microbiology and Genetics, Georg-August-University of Göttingen, 37077 Göttingen, Germany; 2 Institute of Medical Microbiology, University Medical Center Göttingen, 37075 Göttingen, Germany

**Keywords:** Bacterial pathogenesis, Microbiome, Sequencing, Clostridium difficile, Gastroenteritis

## Abstract

We present bacterial 16S rRNA gene datasets derived from stool samples of 44 patients with diarrhea indicative of a *Clostridioides difficile* infection. For 20 of these patients, *C. difficile* infection was confirmed by clinical evidence. Stool samples from patients originating from Germany, Ghana, and Indonesia were taken and subjected to DNA isolation. DNA isolations of stool samples from 35 asymptomatic control individuals were performed. The bacterial community structure was assessed by 16S rRNA gene analysis (V3-V4 region). Metadata from patients and control individuals include gender, age, country, presence of diarrhea, concomitant diseases, and results of microbiological tests to diagnose *C. difficile* presence. We provide initial data analysis and a dataset overview. After processing of paired-end sequencing data, reads were merged, quality-filtered, primer sequences removed, reads truncated to 400 bp and dereplicated. Singletons were removed and sequences were sorted by cluster size, clustered at 97% sequence similarity and chimeric sequences were discarded. Taxonomy to each operational taxonomic unit was assigned by BLASTn searches against Silva database 123.1 and a table was constructed.

## Background & Summary

Infections with *Clostridioides difficile* (formerly *Clostridium difficile*, see Lawson *et al.*^1^) have significantly increased over the past decade^2–5^. The organism is a Gram-positive, obligate anaerobic spore-forming bacterium, which is frequently found as member of the gut microbiome in healthy individuals, but eventually can also act as human pathogen causing disease that ranges from severe diarrhea to life-threatening toxic megacolon^6^. It produces two potent exotoxins, toxin A (enterotoxin, *tcdA*) and toxin B (cytotoxin, *tcdB*)^7^. Some isolates also express a third, so-called binary toxin (*C. difficile* transferase, CDT)^8^. The risk to suffer from a *C. difficile* infection increases with prior broad-spectrum antibiotic treatment, which supports the assumption that an imbalanced gut microbiome increases the likelihood of a *C. difficile* infection^9^.

In this data report, we provide the bacterial community composition in stool samples of 79 human individuals including 44 patients with diarrhea indicative for infection with *C. difficile* and 35 asymptomatic control individuals from regions of Germany (Seesen, Lower Saxony), Ghana (Eikwe, Western Region), and Indonesia (Medan, Sumatra)., For 20 of the 44 patients, clinical evidence of a *C. difficile* infection was obtained. For the remaining patients, the presence of *C. difficile* was indicated by 16S rRNA gene data or MALDI-TOF mass spectrometry. In total, we provide 20,844,594 paired-end 16S rRNA gene reads sequenced with the v3 chemistry of Illumina and a MiSeq instrument. Correspondingly, this dataset represents a total of 10,422,297 bacterial 16S rRNA gene sequences. After all processing steps, which included read-merging, quality-filtering, primer sequence removal, dereplication, singleton removal, read-trimming, chimera removal, and removal of extrinsic domains (Archaea, chloroplasts) 7.204.189 (69.1%) high quality 16S rRNA gene sequences remained for analysis (see Table 1 (available online only) for 16S rRNA gene sequence processing statistics). Additionally, we supply metadata including gender, age, country, presence or absence of diarrhea, *C. difficile* ribotype, toxin PCR ribotype, toxin test from stool, concomitant diseases at time of sampling, and antiobiotic treatment within the last three months (Table 2 (available online only)).

**Figure 1:**
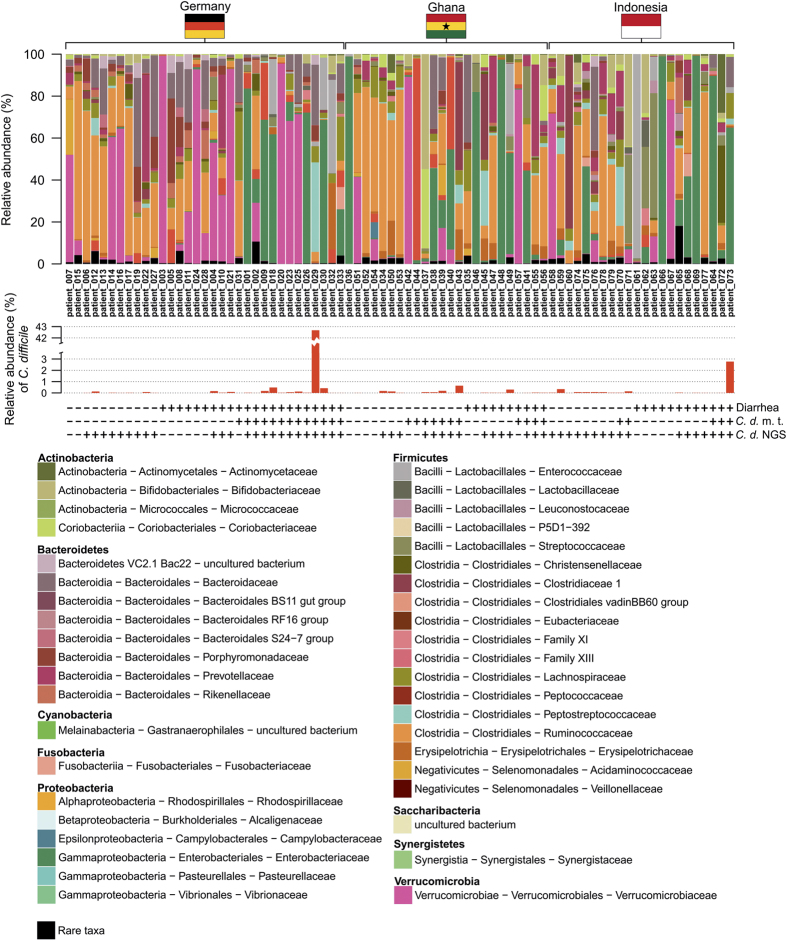
Bacterial community composition at family level of human stool samples analysed in this study. The bacterial community profiles are based on operational taxonomic unit (OTU, defined at 97% genetic identity) frequency in stool samples of 44 patients with diarrhea indicative of *C. difficile* infection and 35 asymptomatic control individuals (*n*=79). One stool sample per patient was used and amplicon PCRs were performed in triplicate for this analysis. Families, which exhibited an abundance of lower than 1% in the entire dataset, were summarized as rare taxa. Relative abundance of *C. difficile* (*Peptoclostridium difficile* in SILVA database 123.1) is displayed separately and exhibited highest similarity to *Clostridioides difficile* strain 630 delta erm (Accession number CP016318). Occurrence of diarrhea in patents is indicated by plus (patient exhibited diarrhea) and minus (no diarrhea), results from microbiological diagnosis of *C. difficile* infection (*C. d.* m. t.) are shown below (plus, positively tested for *C. difficile*; minus, negatively tested for *C. difficile*). Presence and absence of *C. difficile* in amplicon data (*C. d.* NGS) are indicated by plus (present) and minus (absent). Data processing and employed tools are described in detail in the methods section.

The dataset contributes to unveil the significance of the gut microbiome in diseased and asymptomatic patients. In a first analysis, we observed *C. difficile* as a rather low abundant (mainly <1%, with one exception) bacterial community member in stool samples (Fig. 1). The exception was patient_029 (male, age 91), who showed a high abundance of *C. difficile* (42.67%).

Whether the low abundance of *C. difficile* in most stool samples from diarrheic patients might indicate adhesion or invasion of *C. difficile* to the intestinal epithelium remains to be analyzed. However, a similar study also observed low abundances of *C. difficile* in CDI patients^10^. Furthermore, *C. difficile* is not the only potential pathogen of diseased patients. The stool samples of some patients contain other potentially pathogenic bacterial species belonging to different genera such as *Escherichia/Shigella, Salmonella* or *Staphylococcus*. In addition, some stool samples also contained facultative human-pathogenic *Klebsiella* and *Pseudomonas* species. These results support the hypothesis that the gut microbiome contributes to the pathogenic potential or at least can be used as an indicator of *C. difficile* infections. This is of special interest for *C. difficile* infections from Ghana, as most of the so far analyzed genomes of strains from this African country lack the toxin genes^11^. Furthermore, most German patients had a higher age than the patients from the other regions and showed a typical *C. difficile* infection profile, including treatment with antibiotics and presence of mainly toxin-positive strains. In contrast patients from Ghana and Indonesia were younger and had less antibiotic treatment than the German patients, and harboured predominantly toxin-negative strains (Table 2 (available online only)).

**Figure 2:**
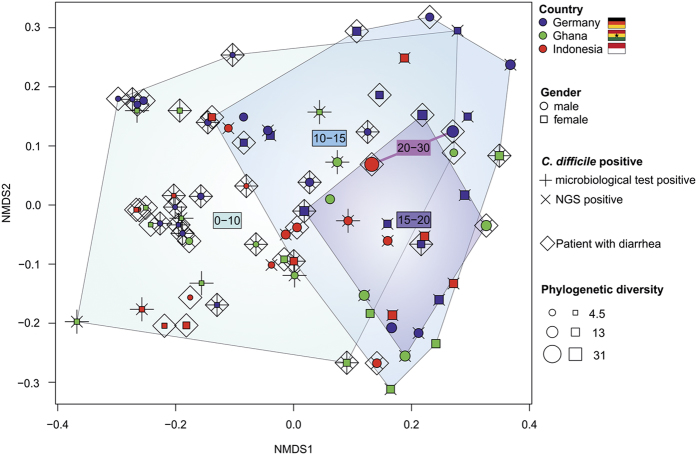
Multivariate analysis of the bacterial community from human stool samples. Non-metric multidimensional scaling (NMDS) based on weighted Unifrac^12^ was used to display the bacterial community structure in 79 stool samples at same sequencing effort (10.000 reads per sample). Samples from patients who exhibited diarrhea at time of sampling are encased by diamond. Samples from patients that were positively tested on *C. difficile* by microbiological test are marked by plus, samples of patients where *C. difficile* was detected in the amplicon dataset are marked by cross. Point size represents the phylogenetic diversity (PD, Faith's Phylogenetic Diversity^26^) of the microbiome, samples are encircled by PD ranges from 0–10, 10–15, 15–20, and 20–30. Data processing and employed tools are described in detail in the methods section. All alpha diversity metrics obtained by QIIME are listed in Table 3 (available online only).

The Unifrac^12^ based bacterial community structure comparison shows variations in structure and diversity within potentially *C. difficile-*infected and reference patients (Fig. 2). We observed a low but significant correlation of the bacterial microbiome to patients who exhibited diarrhea (*P*=0.006, *r*^2^=0.0709) and diagnosed *C. difficile* positive by microbiological tests (*P*=0.017, *r*^2^=0.0628), respectively. In general, patients that have been diagnosed *C. difficile* positive harbour a less diverse bacterial microbiome (Fig. 2), which has also been observed recently^13,14^.

## Methods

### Stool sample preparation and processing

This study was approved by the Ethical Committee of the University Medical Center, Göttingen, Germany (2011-03-29). Diarrhea was defined as the passage of ≥three loose or liquid defecations per day. Upon informed consent, randomly selected patients with diarrhea and non-diarrheal volunteers agreed to submit a stool sample using stool containers and complete a standardised questionnaire about their lifestyle and medical history. Within two hours after providing the stool samples, they were cultured on *Clostridium difficile* agar base used with selective supplement (Oxoid, Basingstoke, Hampshire, UK) and 7% (v/v) defibrinated human blood for 48 h at 38 °C in anaerobic condition using gas packs (bioMérieux, Marcy-l’Ètoile, France). Stool samples were also tested for the presence of *C. difficile* glutamate dehydrogenase (GDH) antigen and toxins A and B by the C. DIFF QUIK CHEK COMPLETE test (Techlab, Blacksburg, USA). In addition, the stool sample that was used for *C. difficile* identification was also frozen immediately after taken from the patients, stored at −20 °C for a maximum of 11 months (based on duration of local sampling period) and transported within 24 h to Göttingen (Germany), where identification of *C. difficile* was confirmed by recultivation and MALDI-TOF mass spectrometry using Biotyper (Bruker Daltonics, Bremen, Germany) with score values of ≥2,000. All *C. difficile* strains were further characterized by toxin determination using the RealStar *Clostridium difficile* PCR Kit 1.0 (Altona Diagnostics, Hamburg, Germany). Ribotyping and toxinotyping was kindly performed by L. von Müller (Homburg, Germany) and M. Rupnik (Maribor, Slovenia) as previously be reported^11^. In addition, the Luminex xTag GPP test was used for all Ghanaian stool samples according to the manufacturer’s instructions (Luminex, Hertogenbosch, The Netherlands) in order to identify *C. difficile* and other potential intestinal pathogens^11^. The stool sample was also used for DNA isolation in order to determine bacterial community composition.

### Nucleic acid extraction and amplification of 16S rRNA genes

DNA was extracted from all stool samples using the MagNA Pure LC 2.0 Instrument with the MagNA Pure LC Total Nucleic Acid Isolation kit following the instructions of the manufacturer (Roche, Mannheim, Germany). Bacterial 16S rRNA gene amplicons were generated using fusion primers TCGTCGGCAGCGTCAGATGTGTATAAGAGACAG-CCTACGGGNGGCWGCAG (MiSeq_overhang-D-Bact-0341-b-S-17) and GTCTCGTGGGCTCGGAGATGTGTATAAGAGACAG-GACTACHVGGGTATCTAATCC (MiSeq_overhang-S-D-Bact-0785-a-A-21) including bacteria targeting primers from Klindworth *et al.*^15^. The PCR reaction mixture with a total volume 50 μl contained 1 U Phusion high fidelity DNA polymerase (Biozym Scientific, Oldendorf, Germany), 5% DMSO, 0.2 mM of each primer, 200 μM dNTP, 0.2 μl of 50 mM MgCl_2_, and 25 ng of isolated DNA. Thermal cycling scheme for bacterial amplicons was as follows: initial denaturation for 1 min at 98 °C, 25 cycles at 98 °C for 45 s, 45 s at 60 °C, and 30 s at 72 °C, and a final extension at 72 °C for 5 min. The resulting PCR products were checked by agarose gel electrophoresis for appropriate size and purified using the magnetic bead capture kit NucleoMag PCR (Macherey-Nagel, Düren, Germany) as recommended by the manufacturer. Quantification of the PCR products was performed using the Quant-iT dsDNA HS assay kit and a Qubit fluorometer (Invitrogen GmbH, Karlsruhe, Germany) following the manufacturer’s instructions. PCR products were used to attach indices and Illumina sequencing adapters using the Nextera XT Index kit (Illumina, San Diego). Index PCR was performed using 5 μl of template PCR product, 2.5 μl of each index primer, 12.5 μl of 2x KAPA HiFi HotStart ReadyMix and 2.5 μl PCR grade water. Thermal cycling scheme was as follows: 95 °C for 3 min, 8 cycles of 30 s at 95 °C, 30 s at 55 °C and 30 s at 72 °C and a final extension at 72 °C for 5 min. Bacterial 16S rRNA genes were sequenced using the dual index paired-end (v3, 2×300 bp) approach for the Illumina MiSeq platform as recommended by the manufacturer.

### 16S rRNA gene sequence processing and analyses

Demultiplexing and clipping of sequence adapters from raw sequences were performed by employing CASAVA data analysis software (Illumina). Paired-end sequences were merged using PEAR v0.9.10^16^ with default parameters. Subsequently, sequences with an average quality score lower than 20 and containing unresolved bases were removed with the *split_libraries_fastq.py* script from QIIME 1.9.1^17^. We additionally removed non-clipped reverse and forward primer sequences by employing cutadapt 1.10^18^ with default settings. For operational taxonomic unit (OTU) clustering, we used USEARCH version 8.1.1861^19^ with the UPARSE^20^ algorithm to truncate reads to 400 bp (-fastx_truncate), dereplicate (-derep_fulllength), sort by cluster size and remove singletons (-sortbysize). Subsequently, OTUs were clustered at 97% sequence identity using USEARCH (-cluster_otus), which includes *de novo* chimera removal. Additionally, chimeric sequences were removed using UCHIME^21^ included in software package USEARCH with reference mode (-uchime_ref) against RDPs trainset15_092015.fasta^22^. All quality-filtered sequences were mapped to chimera-free OTUs and an OTU table was created using USEARCH (-usearch_global). Taxonomic classification of the picked reference sequences (OTUs) was performed with *parallel_assign_taxonomy_blast.py* against SILVA SSU database release 123.1^23^. Extrinsic domain OTUs, chloroplasts, and unclassified OTUs were removed from the dataset by employing *filter_otu_table.py*. Sample comparisons were performed at the same surveying effort, utilizing the lowest number of sequences by random resampling (10.000 reads per sample). Species richness, alpha and beta diversity estimates were determined using the QIIME script *alpha*_*rarefaction*.*py*. Non-metric multidimensional scaling (NMDS) and statistical tests were performed with the vegan package^24^ in R^25^.

## Data Records

The paired-end reads of the 16S rRNA gene sequencing were deposited in the National Center for Biotechnology Information (Data Citation 1). The dataset consists of 158 zipped FASTQ files that were processed by the CASAVA software (Illumina), which includes demultiplexing and removal of adapter sequences. The OTU table (otu_table_PRJNA353065.xlsx) used for all analyses and the corresponding representative OTU sequences clustered at 97% genetic identity (otu_sequences_PRJNA353065.fasta) are accessible at figshare.com (Data Citation 2).

## Technical Validation

Success of 16S rRNA gene amplicon generation was controlled by reviewing the amplicon size (approximately 550 bp) and absence of contaminations on an agarose gel. Additionally, negative (PCR reaction without template) and positive controls (genomic DNA of *E. coli* DH5a) were performed to ensure purity of the employed reagents. To reduce possible PCR biases, all PCRs were performed in triplicate and after purification pooled equimolar.

## Usage Notes

The OTU table (otu_table_PRJNA353065.xlsx) used for all analyses and the corresponding representative OTU sequences clustered at 97% genetic identity (otu_sequences_PRJNA353065.fasta) are accessible at figshare (Data Citation 2).

## Additional Information

**How to cite this article:** Schneider, D. *et al.* Gut bacterial communities of diarrheic patients with indications of *Clostridioides difficile* infection. *Sci. Data* 4:170152 doi: 10.1038/sdata.2017.152 (2017).

**Publisher’s note:** Springer Nature remains neutral with regard to jurisdictional claims in published maps and institutional affiliations.

## Supplementary Material



## Figures and Tables

**Table 1:** 16S rRNA gene sequence processing statistics and biosample accession numbers

**Sample name**	**Biosample accession**	**Raw reads (2x, paired-end)**	**After PEAR**	% loss*	**After QF**	**% loss** *	**After taxonomy filter**	**% loss** *
patient_001	SAMN06011251	183,885	178,258	3.06	128,097	30.3	91,562	50.2
patient_002	SAMN06011252	283,254	272,331	3.86	208,965	26.2	189,871	33.0
patient_003	SAMN06011253	29,313	28,372	3.21	24,528	16.3	23,857	18.6
patient_004	SAMN06011254	19,854	18,967	4.47	16,185	18.5	14,889	25.0
patient_005	SAMN06011255	77,418	73,960	4.47	60,097	22.4	56,675	26.8
patient_006	SAMN06011256	118,110	112,829	4.47	92,535	21.7	73,601	37.7
patient_007	SAMN06011257	61,760	57,107	7.53	46,348	25.0	44,385	28.1
patient_008	SAMN06011258	188,584	183,121	2.90	160,489	14.9	151,302	19.8
patient_009	SAMN06011259	121,488	116,373	4.21	76,062	37.4	72,353	40.4
patient_010	SAMN06011260	47,754	45,826	4.04	38,386	19.6	36,312	24.0
patient_011	SAMN06011261	1,137,384	1,099,480	3.33	921,087	19.0	856,364	24.7
patient_012	SAMN06011262	29,601	28,353	4.22	23,893	19.3	20,824	29.7
patient_013	SAMN06011263	603,255	575,795	4.55	470,894	21.9	419,480	30.5
patient_014	SAMN06011264	27,890	26,906	3.53	22,664	18.7	21,306	23.6
patient_015	SAMN06011265	87,995	85,717	2.59	72,649	17.4	67,572	23.2
patient_016	SAMN06011266	81,374	77,871	4.30	65,934	19.0	56,602	30.4
patient_017	SAMN06011267	39,762	37,969	4.51	31,481	20.8	28,623	28.0
patient_018	SAMN06011268	69,262	66,730	3.66	25,195	63.6	23,399	66.2
patient_019	SAMN06011269	83,424	80,839	3.10	67,259	19.4	63,697	23.6
patient_020	SAMN06011270	108,817	104,597	3.88	89,081	18.1	85,928	21.0
patient_021	SAMN06011271	123,228	118,815	3.58	101,485	17.6	97,888	20.6
patient_022	SAMN06011272	27,398	26,520	3.20	21,818	20.4	19,523	28.7
patient_023	SAMN06011273	782,019	752,476	3.78	635,823	18.7	605,208	22.6
patient_024	SAMN06011274	570,482	548,037	3.93	466,962	18.1	450,550	21.0
patient_025	SAMN06011275	29,420	28,252	3.97	23,626	19.7	22,380	23.9
patient_026	SAMN06011276	58,090	55,621	4.25	26,390	54.6	24,952	57.0
patient_027	SAMN06011277	29,432	28,109	4.50	22,539	23.4	20,812	29.3
patient_028	SAMN06011278	181,138	173,803	4.05	140,608	22.4	126,714	30.0
patient_029	SAMN06011279	41,796	40,543	3.00	35,367	15.4	33,444	20.0
patient_030	SAMN06011280	45,345	43,748	3.52	30,554	32.6	28,364	37.4
patient_031	SAMN06011281	48,745	47,545	2.46	13,517	72.3	12,569	74.2
patient_032	SAMN06011282	50,655	48,039	5.16	21,797	57.0	20,248	60.0
patient_033	SAMN06011283	109,639	105,711	3.58	75,174	31.4	61,098	44.3
patient_034	SAMN06011284	19,449	18,806	3.31	16,053	17.5	14,558	25.1
patient_035	SAMN06011285	45,502	43,883	3.56	36,311	20.2	31,231	31.4
patient_036	SAMN06011286	112,545	107,687	4.32	80,577	28.4	78,582	30.2
patient_037	SAMN06011287	26,917	25,454	5.44	20,435	24.1	19,494	27.6
patient_038	SAMN06011288	43,597	42,515	2.48	34,868	20.0	33,540	23.1
patient_039	SAMN06011289	117,718	114,512	2.72	97,788	16.9	39,043	66.8
patient_040	SAMN06011290	73,905	69,618	5.80	54,943	25.7	52,717	28.7
patient_041	SAMN06011291	40,730	39,301	3.51	32,688	19.7	31,371	23.0
patient_042	SAMN06011292	38,409	37,155	3.26	31,748	17.3	29,962	22.0
patient_043	SAMN06011293	13,346	13,007	2.54	11,381	14.7	10,650	20.2
patient_044	SAMN06011294	16,592	15,995	3.60	13,586	18.1	13,207	20.4
patient_045	SAMN06011295	77,488	74,526	3.82	62,027	20.0	57,396	25.9
patient_046	SAMN06011296	66,527	64,443	3.13	51,997	21.8	50,861	23.5
patient_047	SAMN06011297	131,863	129,034	2.15	110,888	15.9	104,209	21.0
patient_048	SAMN06011298	27,782	27,214	2.04	22,383	19.4	22,103	20.4
patient_049	SAMN06011299	18,338	17,774	3.08	14,603	20.4	13,978	23.8
patient_050	SAMN06011300	47,408	46,252	2.44	39,412	16.9	36,086	23.9
patient_051	SAMN06011301	120,937	117,898	2.51	102,904	14.9	95,667	20.9
patient_052	SAMN06011302	59,282	57,527	2.96	49,677	16.2	47,377	20.1
patient_053	SAMN06011303	78,974	77,534	1.82	67,320	14.8	62,539	20.8
patient_054	SAMN06011304	35,543	34,385	3.26	28,947	18.6	24,646	30.7
patient_055	SAMN06011305	95,179	92,620	2.69	75,463	20.7	70,818	25.6
patient_056	SAMN06011306	27,168	26,425	2.73	22,551	17.0	21,338	21.5
patient_057	SAMN06011307	74,445	72,231	2.97	62,599	15.9	60,439	18.8
patient_058	SAMN06011308	329,680	317,312	3.75	268,820	18.5	257,896	21.8
patient_059	SAMN06011309	21,033	20,266	3.65	16,779	20.2	15,706	25.3
patient_060	SAMN06011310	432,732	421,277	2.65	352,201	18.6	336,711	22.2
patient_061	SAMN06011311	24,320	23,479	3.46	18,993	21.9	18,224	25.1
patient_062	SAMN06011312	38,558	35,981	6.68	28,868	25.1	27,283	29.2
patient_063	SAMN06011313	56,156	51,606	8.10	41,663	25.8	38,647	31.2
patient_064	SAMN06011314	46,732	44,780	4.18	34,617	25.9	33,399	28.5
patient_065	SAMN06011315	66,685	64,446	3.36	52,337	21.5	44,968	32.6
patient_066	SAMN06011316	133,075	128,615	3.35	100,317	24.6	98,069	26.3
patient_067	SAMN06011317	172,365	166,723	3.27	142,932	17.1	137,226	20.4
patient_068	SAMN06011318	70,140	68,535	2.29	59,169	15.6	55,802	20.4
patient_069	SAMN06011319	38,007	37,056	2.50	30,107	20.8	29,577	22.2
patient_070	SAMN06011320	13,530	13,126	2.99	11,143	17.6	10,443	22.8
patient_071	SAMN06011321	163,830	155,275	5.22	126,207	23.0	118,940	27.4
patient_072	SAMN06011322	82,488	79,151	4.05	64,796	21.4	59,576	27.8
patient_073	SAMN06011323	1,421,967	1,376,514	3.20	1,126,249	20.8	676,497	52.4
patient_074	SAMN06011324	122,784	117,609	4.21	96,116	21.7	86,400	29.6
patient_075	SAMN06011325	18,848	18,218	3.34	14,945	20.7	14,161	24.9
patient_076	SAMN06011326	34,771	33,688	3.11	27,875	19.8	26,132	24.8
patient_077	SAMN06011327	69,092	66,520	3.72	55,769	19.3	50,121	27.5
patient_078	SAMN06011328	113,883	111,135	2.41	98,305	13.7	92,503	18.8
patient_079	SAMN06011329	44,406	42,365	4.60	34,012	23.4	31,744	28.5

*calculated from raw reads.

**Table 2:** Metadata of patients

**Sample name**	**C. difficile culture**	**Ribotype**	**Toxin PCR Ribotype**	**Toxin from stool (GDH)**	***C. difficile*** **detection by NGS**	**Conformity of** ***C. difficil*****e detection**	**Country**	**Gender**	**Age (y)**	**diarrhea**	**Concomitant diseases**	**Antibiotics last three months**
patient_001	positive	27	A+B+CDT+	positive	positive	true	Germany	male	75	yes	none	yes
patient_002	positive	001/072	A+B+CDT−	positive	positive	true	Germany	female	79	yes	none	no
patient_003	negative	NA	NA	NA	negative	true	Germany	male	87	yes	none	yes
patient_004	negative	NA	NA	NA	positive	false	Germany	female	93	yes	none	yes
patient_005	negative	NA	NA	NA	negative	true	Germany	male	75	yes	none	yes
patient_006	negative	NA	NA	NA	positive	false	Germany	female	62	no	none	no
patient_007	negative	NA	NA	NA	negative	true	Germany	male	49	no	none	yes
patient_008	negative	NA	NA	NA	negative	true	Germany	male	69	yes	none	no
patient_009	positive	001/072	A+B+CDT−	negative	positive	true	Germany	male	77	yes	none	yes
patient_010	negative	NA	NA	NA	positive	false	Germany	female	63	yes	none	no
patient_011	negative	NA	NA	NA	negative	true	Germany	female	64	yes	none	no
patient_012	negative	NA	NA	NA	positive	false	Germany	female	75	no	none	no
patient_013	negative	NA	NA	NA	positive	false	Germany	female	75	no	none	no
patient_014	negative	NA	NA	NA	positive	false	Germany	female	65	no	none	no
patient_015	negative	NA	NA	NA	negative	true	Germany	male	71	no	none	yes
patient_016	negative	NA	NA	NA	positive	false	Germany	male	79	no	none	no
patient_017	negative	NA	NA	NA	positive	false	Germany	male	56	no	none	no
patient_018	positive	001/072	A+B+CDT−	negative	positive	true	Germany	male	77	yes	noro virus	yes
patient_019	negative	NA	NA	NA	positive	false	Germany	female	31	no	none	no
patient_020	positive	78	A+B+CDT+	negative	positive	true	Germany	male	57	yes	noro virus	yes
patient_021	negative	NA	NA	NA	positive	false	Germany	male	85	yes	none	yes
patient_022	negative	NA	NA	NA	positive	false	Germany	male	84	no	none	yes
patient_023	positive	78	A+B+CDT+	negative	positive	true	Germany	male	57	yes	noro virus	yes
patient_024	negative	NA	NA	NA	negative	true	Germany	male	80	yes	noro virus	yes
patient_025	positive	78	A+B+CDT+	negative	positive	true	Germany	male	59	yes	none	yes
patient_026	positive	001/072	A+B+CDT−	negative	positive	true	Germany	male	82	yes	none	yes
patient_027	negative	NA	NA	NA	positive	false	Germany	female	81	no	none	NA
patient_028	negative	NA	NA	NA	negative	true	Germany	female	72	yes	none	yes
patient_029	positive	2	A+B+CDT−	positive	positive	true	Germany	male	91	yes	none	yes
patient_030	positive	001/072	A+B+CDT−	positive	positive	true	Germany	female	84	yes	none	yes
patient_031	positive	78	A+B+CDT+	negative	negative	true	Germany	female	85	yes	noro virus	yes
patient_032	positive	2	A+B+CDT−	negative	positive	true	Germany	female	70	yes	none	yes
patient_033	positive	001/072	A+B+CDT−	negative	positive	true	Germany	male	73	yes	none	yes
patient_034	negative	NA	NA	NA	positive	false	Ghana	male	45	no	none	Amoxicillin
patient_035	negative	NA	NA	NA	negative	true	Ghana	male	47	yes	suspected cholera	no
patient_036	negative	NA	NA	NA	negative	true	Ghana	male	9	no	acute abdomen (suspected typhoid)	no
patient_037	positive	SLO 233	A+B+CDT−	positive	positive	true	Ghana	female	1.17	no	ETEC/LT	Cloxacillin
patient_038	positive	084 (CE)	A−B−CDT−	negative	positive	true	Ghana	female	2	no	ETEC/LT	Amoxicillin
patient_039	positive	SLO 095	A−B−CDT−	negative	positive	true	Ghana	male	72	no	none	no
patient_040	positive	011/049	A−B−CDT−	negative	positive	true	Ghana	female	30	no	acute abdomen	no
patient_041	positive	SLO091	A−B−CDT−	negative	positive	true	Ghana	male	1.17	yes	malaria, rota virus, ETEC/LT	no
patient_042	positive	097 (CE)	A−B−CDT−	negative	negative	true	Ghana	female	24	no	none	Metronidazole, Ciprofloxacin
patient_043	positive	084 (CE)	A−B−CDT−	negative	positive	true	Ghana	male	5	no	Giardia	no
patient_044	positive	SLO 235	A−B−CDT−	negative	negative	true	Ghana	female	0.67	no	Shigella	no
patient_045	negative	NA	NA	NA	positive	false	Ghana	female	34	yes	none	no
patient_046	negative	NA	NA	NA	negative	true	Ghana	female	1.42	yes	malaria, chest infection	no
patient_047	negative	NA	NA	NA	positive	false	Ghana	male	44	yes	none	no
patient_048	negative	NA	NA	NA	positive	false	Ghana	female	56	yes	chest infection	Amoxicillin in the last 6 months
patient_049	negative	NA	NA	NA	positive	false	Ghana	male	84	yes	none	Metronidazole, Ampicillin
patient_050	negative	NA	NA	NA	positive	false	Ghana	male	52	no	none	Metronidazole in the last 6 months
patient_051	negative	NA	NA	NA	negative	true	Ghana	male	78	no	none	no
patient_052	negative	NA	NA	NA	negative	true	Ghana	female	41	no	none	no
patient_053	negative	NA	NA	NA	positive	false	Ghana	female	28	no	none	no
patient_054	negative	NA	NA	NA	negative	true	Ghana	female	72	no	none	no
patient_055	positive	SLO 233	A+B+CDT−	positive	positive	true	Ghana	female	22	yes	Campylobacter	no
patient_056	positive	084 (CE)	A−B−CDT−	negative	positive	true	Ghana	female	40	yes	none	Metronidazole
patient_057	positive	084 (CE)	A−B−CDT−	negative	negative	true	Ghana	female	1.33	yes	malaria, Campylobacter	no
patient_058	negative	NA	NA	NA	positive	false	Indonesia	male	3	no	none	no
patient_059	negative	NA	NA	NA	positive	false	Indonesia	male	13	no	none	no
patient_060	negative	NA	NA	NA	positive	false	Indonesia	male	65	no	none	no
patient_061	negative	NA	NA	NA	negative	true	Indonesia	male	12	yes	none	no
patient_062	negative	NA	NA	NA	negative	true	Indonesia	female	3	yes	none	no
patient_063	negative	NA	NA	NA	negative	true	Indonesia	female	20	yes	none	no
patient_064	positive	17	A−B+CDT−	negative	positive	true	Indonesia	female	56	yes	none	no
patient_065	negative	NA	NA	NA	positive	false	Indonesia	male	1	yes	none	no
patient_066	negative	NA	NA	NA	negative	true	Indonesia	female	70	yes	none	no
patient_067	negative	NA	NA	NA	negative	true	Indonesia	female	26	yes	none	no
patient_068	negative	NA	NA	NA	positive	false	Indonesia	male	46	yes	none	no
patient_069	negative	NA	NA	NA	positive	false	Indonesia	female	53	yes	none	no
patient_070	positive	53	A+B+CDT−	negative	positive	true	Indonesia	male	44	no	none	no
patient_071	positive	SLO 236	A+B+CDT−	positive	positive	true	Indonesia	female	0.75	no	none	no
patient_072	positive	SLO 160	A+B+CDT−	negative	positive	true	Indonesia	female	10	yes	none	no
patient_073	positive	10	A−B−CDT−	negative	positive	true	Indonesia	male	0.16	yes	none	no
patient_074	negative	NA	NA	NA	positive	false	Indonesia	female	11	no	none	no
patient_075	negative	NA	NA	NA	positive	false	Indonesia	male	100	no	none	no
patient_076	negative	NA	NA	NA	positive	false	Indonesia	female	85	no	none	no
patient_077	negative	NA	NA	NA	positive	false	Indonesia	male	67	yes	none	no
patient_078	negative	NA	NA	NA	positive	false	Indonesia	female	41	no	none	no
patient_079	negative	NA	NA	NA	positive	false	Indonesia	female	49	no	none	no

**Table 3:** Diversity metrics for each bacterial microbiome at a sequence depth of 10.000 16S rRNA gene reads

**Sample name**	**PD_whole_tree**	**berger_parker_d**	**brillouin_d**	**chao1**	**chao1_lower_bound**	**chao1_upper_bound**	**dominance**	**doubles**	**enspie**	**equitability**	**esty_lower_bound**	**esty_upper_bound**	**fisher_alpha**	**gini_index**	**goods_coverage**	**heip_e**	**margalef**	**mcintosh_d**	**mcintosh_e**	**menhinick**	**michaelis_menten_fit**	**observed**	**observed_otus**	**observed_species**	**robbins**	**shannon**	**simpson**	**simpson_e**	**simpson_reciprocal**	**singles**	**strong**
patient_001	4.161	0.835	0.807	47.749	37.729	89.166	0.702	3.5	1.425	0.23	0	0.002	4.463	0.998	0.999	0.038	3.626	0.164	0.84	0.344	36.036	34.4	34.4	34.4	0.001	1.173	0.298	0.042	1.425	9.7	0.824
patient_002	16.147	0.184	3.472	299.526	268.222	358.724	0.064	29.9	15.735	0.645	0.004	0.009	42.671	0.976	0.994	0.14	25.189	0.755	0.258	2.33	261.928	233	233	233	0.006	5.069	0.936	0.068	15.735	63.6	0.739
patient_003	4.448	0.988	0.086	59.046	33.798	149.066	0.976	2.7	1.024	0.028	0.001	0.002	2.942	0.998	0.998	0.004	2.486	0.012	0.99	0.239	38.576	23.9	23.9	23.9	0.002	0.129	0.024	0.043	1.024	15.1	0.946
patient_004	10.454	0.562	2.185	152.063	135.923	192.864	0.328	13.8	3.047	0.457	0.001	0.004	20.186	0.989	0.997	0.065	13.496	0.431	0.58	1.253	133.141	125.3	125.3	125.3	0.003	3.187	0.672	0.024	3.047	27.8	0.744
patient_005	10.483	0.383	2.476	160.903	137.006	216.238	0.186	14.9	5.369	0.523	0.002	0.005	18.931	0.99	0.996	0.095	12.779	0.574	0.437	1.187	132.337	118.7	118.7	118.7	0.004	3.604	0.814	0.045	5.369	35.3	0.765
patient_006	14.41	0.352	3.016	229.708	206.752	278.419	0.145	21.4	6.921	0.584	0.003	0.006	32.481	0.981	0.996	0.109	20.108	0.626	0.387	1.862	202.688	186.2	186.2	186.2	0.004	4.402	0.855	0.037	6.921	44.2	0.731
patient_007	10.589	0.509	1.739	164.959	139.239	222.362	0.334	17	2.993	0.369	0.002	0.006	18.854	0.994	0.996	0.041	12.736	0.426	0.585	1.183	138.591	118.3	118.3	118.3	0.004	2.538	0.666	0.025	2.993	39.2	0.841
patient_008	21.359	0.135	3.684	355.093	319.288	418.096	0.052	39.6	19.412	0.666	0.006	0.011	51.622	0.97	0.992	0.15	29.434	0.781	0.233	2.721	310.865	272.1	272.1	272.1	0.008	5.384	0.948	0.071	19.412	81.2	0.726
patient_009	4.886	0.415	1.549	52.88	44.309	85.316	0.283	5.6	3.531	0.419	0	0.002	5.489	0.996	0.999	0.093	4.365	0.473	0.534	0.412	43.804	41.2	41.2	41.2	0.001	2.246	0.717	0.086	3.531	12.1	0.838
patient_010	13.716	0.338	2.297	220.044	195.354	271.431	0.222	22.2	4.509	0.452	0.003	0.007	29.598	0.989	0.995	0.054	18.61	0.534	0.479	1.724	199.008	172.4	172.4	172.4	0.005	3.356	0.778	0.026	4.509	47	0.806
patient_011	11.525	0.241	3.042	202.257	170.498	270.235	0.09	17.8	11.079	0.619	0.003	0.006	23.561	0.986	0.995	0.145	15.374	0.707	0.305	1.426	156.56	142.6	142.6	142.6	0.005	4.427	0.91	0.078	11.079	45.9	0.751
patient_012	10.93	0.358	2.874	142.962	132.508	172.753	0.15	12.7	6.682	0.599	0.001	0.003	20.475	0.985	0.998	0.137	13.659	0.619	0.392	1.268	133.399	126.8	126.8	126.8	0.002	4.183	0.85	0.053	6.682	21	0.703
patient_013	14.921	0.203	3.422	262.995	231.609	324.168	0.068	26	14.711	0.654	0.004	0.008	35.082	0.978	0.994	0.156	21.432	0.747	0.266	1.984	219.964	198.4	198.4	198.4	0.006	4.989	0.932	0.074	14.711	58	0.719
patient_014	12.575	0.585	2.179	197.939	176.597	243.469	0.35	22.7	2.86	0.436	0.002	0.006	26.582	0.988	0.996	0.052	17.013	0.413	0.601	1.577	175.998	157.7	157.7	157.7	0.004	3.184	0.65	0.018	2.86	42.9	0.767
patient_015	15.965	0.223	3.168	256.497	235.69	298.721	0.089	29.7	11.3	0.597	0.003	0.007	38.767	0.98	0.995	0.111	23.278	0.71	0.304	2.154	245.062	215.4	215.4	215.4	0.005	4.627	0.911	0.052	11.3	49.5	0.764
patient_016	12.112	0.64	1.739	185.118	159.595	240.294	0.424	18.5	2.358	0.358	0.002	0.006	22.558	0.992	0.996	0.035	14.82	0.352	0.66	1.375	158.988	137.5	137.5	137.5	0.004	2.542	0.576	0.017	2.358	42	0.822
patient_017	12.682	0.253	2.65	190.523	165.267	247.519	0.144	15.5	6.928	0.538	0.002	0.005	24.055	0.988	0.996	0.094	15.645	0.626	0.385	1.451	160.459	145.1	145.1	145.1	0.004	3.861	0.856	0.048	6.928	38.4	0.777
patient_018	5.176	0.521	1.71	68.052	57.194	105.865	0.319	6	3.131	0.434	0	0.002	7.312	0.996	0.999	0.089	5.624	0.439	0.568	0.528	56.304	52.8	52.8	52.8	0.001	2.482	0.681	0.059	3.131	14.3	0.782
patient_019	8.764	0.238	2.75	117.019	101.269	160.077	0.101	10	9.898	0.612	0.001	0.004	14.017	0.99	0.998	0.164	9.891	0.689	0.321	0.921	100.038	92.1	92.1	92.1	0.002	3.994	0.899	0.108	9.898	23.4	0.762
patient_020	4.055	0.954	0.294	46.937	38.359	81.853	0.91	3.7	1.099	0.084	0	0.002	4.625	0.998	0.999	0.01	3.746	0.047	0.957	0.355	39.789	35.5	35.5	35.5	0.001	0.433	0.09	0.031	1.099	10.3	0.925
patient_021	9.485	0.926	0.491	145.711	117.66	209.04	0.858	15	1.165	0.111	0.002	0.006	14.58	0.997	0.996	0.007	10.228	0.074	0.935	0.952	126.8	95.2	95.2	95.2	0.004	0.728	0.142	0.012	1.165	39.3	0.927
patient_022	14.866	0.506	2.601	236.33	215.697	279.414	0.265	25.9	3.779	0.499	0.003	0.007	34.712	0.982	0.995	0.066	21.248	0.49	0.525	1.967	218.421	196.7	196.7	196.7	0.005	3.805	0.735	0.019	3.779	45.7	0.739
patient_023	6.892	0.68	1.482	100.411	85.57	143.617	0.475	9.1	2.107	0.344	0.001	0.003	11.477	0.995	0.998	0.045	8.328	0.314	0.694	0.777	85.434	77.7	77.7	77.7	0.002	2.159	0.525	0.027	2.107	19.9	0.814
patient_024	7.891	0.927	0.46	111.954	86.239	178.008	0.86	9.4	1.163	0.111	0.002	0.004	10.11	0.998	0.997	0.009	7.459	0.074	0.934	0.697	92.57	69.7	69.7	69.7	0.003	0.679	0.14	0.017	1.163	28.9	0.913
patient_025	4.528	0.711	1.133	52.104	41.701	91.291	0.525	4.4	1.904	0.314	0	0.002	4.985	0.997	0.999	0.058	4.006	0.278	0.727	0.379	40.649	37.9	37.9	37.9	0.001	1.645	0.475	0.05	1.904	12.3	0.829
patient_026	6.806	0.706	1.384	82.669	70.913	120.618	0.507	7.6	1.973	0.334	0.001	0.003	9.413	0.996	0.998	0.047	7.014	0.291	0.717	0.656	71.306	65.6	65.6	65.6	0.002	2.015	0.493	0.03	1.973	16.4	0.812
patient_027	10.591	0.203	2.837	158.235	136.719	208.113	0.096	15.5	10.455	0.597	0.002	0.005	19.232	0.988	0.997	0.138	12.953	0.698	0.313	1.203	130.374	120.3	120.3	120.3	0.003	4.126	0.904	0.087	10.455	34.8	0.77
patient_028	17.767	0.17	3.351	340.553	300.292	412.477	0.078	33.6	12.757	0.615	0.005	0.01	46.283	0.976	0.992	0.116	26.926	0.727	0.287	2.49	284.865	249	249	249	0.008	4.898	0.922	0.051	12.757	78	0.734
patient_029	7.401	0.425	2.131	80.851	70.88	115.381	0.224	6.2	4.456	0.51	0	0.002	9.615	0.994	0.999	0.115	7.144	0.532	0.477	0.668	70.403	66.8	66.8	66.8	0.001	3.094	0.776	0.067	4.456	14.2	0.763
patient_030	3.95	0.569	1.363	45.325	36.569	84.81	0.391	2.5	2.558	0.389	0	0.002	4.4	0.997	0.999	0.089	3.583	0.379	0.627	0.34	35.406	34	34	34	0.001	1.977	0.609	0.075	2.558	8.2	0.789
patient_031	11.078	0.208	3.178	184.15	161.285	235.493	0.08	16.7	12.45	0.646	0.002	0.005	23.618	0.983	0.996	0.167	15.407	0.724	0.287	1.429	151.223	142.9	142.9	142.9	0.004	4.625	0.92	0.087	12.45	38.3	0.704
patient_032	4.8	0.409	1.667	42.334	38.249	61.665	0.277	5	3.611	0.464	0	0.002	4.865	0.996	0.999	0.121	3.92	0.479	0.528	0.371	39.758	37.1	37.1	37.1	0.001	2.416	0.723	0.098	3.611	8.1	0.767
patient_033	10.956	0.221	3.159	166.344	142.767	226.439	0.085	10	11.719	0.657	0.001	0.004	20.576	0.984	0.997	0.184	13.713	0.715	0.296	1.273	131.58	127.3	127.3	127.3	0.003	4.595	0.915	0.092	11.719	28.2	0.686
patient_034	16.228	0.46	2.725	254.457	229.63	305.862	0.224	22.7	4.456	0.518	0.003	0.007	36.781	0.981	0.995	0.072	22.29	0.532	0.484	2.063	228.914	206.3	206.3	206.3	0.005	3.986	0.776	0.022	4.456	47.6	0.747
patient_035	9.666	0.237	2.849	131.463	121.285	160.903	0.117	11.9	8.514	0.605	0.001	0.003	18.381	0.987	0.998	0.145	12.464	0.664	0.347	1.158	121.658	115.8	115.8	115.8	0.002	4.144	0.883	0.074	8.514	20.2	0.718
patient_036	2.413	0.934	0.286	20.667	15.705	47.238	0.876	2	1.141	0.109	0	0.001	1.67	0.998	0.999	0.026	1.466	0.065	0.937	0.145	17.858	14.5	14.5	14.5	0.001	0.415	0.124	0.081	1.141	6.2	0.864
patient_037	5.802	0.375	1.482	66.58	52.916	114.969	0.3	4.7	3.332	0.386	0	0.002	6.469	0.996	0.999	0.074	5.049	0.457	0.55	0.475	51.858	47.5	47.5	47.5	0.001	2.15	0.7	0.07	3.332	14.5	0.851
patient_038	5.944	0.413	1.766	63.484	55.717	93.395	0.278	5.5	3.596	0.448	0	0.002	7.329	0.995	0.999	0.095	5.635	0.477	0.53	0.529	54.992	52.9	52.9	52.9	0.001	2.563	0.722	0.068	3.596	11.1	0.786
patient_039	12.45	0.158	3.149	202.605	181.765	249.729	0.081	19.2	12.311	0.623	0.002	0.005	28.111	0.983	0.996	0.14	17.828	0.722	0.29	1.652	179.838	165.2	165.2	165.2	0.004	4.588	0.919	0.075	12.311	37.2	0.734
patient_040	3.87	0.472	1.169	42.485	30.804	90.53	0.393	2.1	2.546	0.357	0	0.002	3.364	0.997	0.999	0.087	2.812	0.377	0.628	0.269	28.188	26.9	26.9	26.9	0.001	1.694	0.607	0.095	2.546	8.4	0.834
patient_041	5.024	0.428	1.699	57.243	46.02	99.753	0.264	4.4	3.785	0.457	0	0.002	5.597	0.996	0.999	0.111	4.441	0.491	0.516	0.419	45.912	41.9	41.9	41.9	0.001	2.463	0.736	0.091	3.785	11.7	0.779
patient_042	4.388	0.891	0.577	34.832	31.55	52.981	0.797	3.6	1.255	0.17	0	0.001	3.928	0.998	0.999	0.027	3.235	0.108	0.895	0.308	32.02	30.8	30.8	30.8	0.001	0.841	0.203	0.041	1.255	6.1	0.869
patient_043	7.855	0.46	2.118	116.103	86.772	212.871	0.246	3	4.059	0.496	0.001	0.003	10.837	0.993	0.998	0.102	7.926	0.509	0.5	0.74	77.03	74	74	74	0.002	3.077	0.754	0.055	4.059	18.4	0.766
patient_044	4.648	0.958	0.274	42.186	36.484	66.469	0.918	5.6	1.09	0.079	0	0.002	4.504	0.998	0.999	0.01	3.659	0.042	0.961	0.347	39.692	34.7	34.7	34.7	0.001	0.404	0.082	0.031	1.09	9.5	0.929
patient_045	9.217	0.403	2.118	121.98	100.456	179.741	0.236	9.3	4.23	0.477	0.001	0.004	13.213	0.993	0.997	0.086	9.402	0.519	0.49	0.876	93.587	87.6	87.6	87.6	0.003	3.079	0.764	0.048	4.23	25.6	0.774
patient_046	4.106	0.82	0.63	42.963	30.003	91.145	0.691	3.6	1.448	0.197	0	0.002	3.124	0.998	0.999	0.037	2.627	0.171	0.833	0.252	33.762	25.2	25.2	25.2	0.001	0.914	0.309	0.058	1.448	13	0.871
patient_047	16.292	0.28	2.767	247.855	216.74	308.925	0.133	24.8	7.524	0.537	0.004	0.008	32.063	0.986	0.994	0.084	19.891	0.642	0.371	1.842	210.583	184.2	184.2	184.2	0.006	4.038	0.867	0.041	7.524	56.1	0.771
patient_048	4.398	0.972	0.181	39.23	29.349	80.428	0.945	3.1	1.058	0.057	0	0.002	3.265	0.998	0.999	0.008	2.736	0.028	0.975	0.262	31.777	26.2	26.2	26.2	0.001	0.266	0.055	0.041	1.058	10	0.934
patient_049	7.766	0.328	1.895	88.194	77.719	119.936	0.232	10.4	4.315	0.446	0.001	0.003	10.582	0.995	0.998	0.08	7.763	0.524	0.485	0.725	81.659	72.5	72.5	72.5	0.002	2.753	0.768	0.06	4.315	18.2	0.823
patient_050	16.041	0.166	3.375	238.375	213.839	289.825	0.069	21.5	14.485	0.649	0.003	0.006	33.583	0.978	0.995	0.154	20.672	0.745	0.268	1.914	206.625	191.4	191.4	191.4	0.005	4.921	0.931	0.076	14.485	46.2	0.712
patient_051	12.965	0.411	2.695	193.632	172.897	239.501	0.188	19.6	5.307	0.54	0.002	0.006	26.179	0.986	0.996	0.092	16.796	0.572	0.441	1.557	171.619	155.7	155.7	155.7	0.004	3.929	0.812	0.034	5.307	39.4	0.754
patient_052	14.094	0.372	2.561	203.307	178.744	256.204	0.184	19.8	5.426	0.512	0.002	0.006	26.506	0.988	0.996	0.079	16.97	0.576	0.436	1.573	175.806	157.3	157.3	157.3	0.004	3.735	0.816	0.035	5.426	42.6	0.772
patient_053	12.022	0.281	2.843	186.495	165.239	233.363	0.121	19.1	8.268	0.575	0.002	0.006	24.534	0.987	0.996	0.114	15.906	0.659	0.353	1.475	161.067	147.5	147.5	147.5	0.004	4.141	0.879	0.056	8.268	39.5	0.758
patient_054	13.052	0.244	2.994	205.205	186.265	246.502	0.101	22.8	9.951	0.589	0.002	0.006	29.139	0.985	0.996	0.116	18.371	0.69	0.322	1.702	191.037	170.2	170.2	170.2	0.004	4.365	0.899	0.058	9.951	40.6	0.771
patient_055	13.858	0.436	2.705	239.039	205.412	306.875	0.205	20.6	4.887	0.532	0.003	0.007	29.555	0.985	0.995	0.084	18.588	0.553	0.46	1.722	190.916	172.2	172.2	172.2	0.005	3.948	0.795	0.028	4.887	52.8	0.749
patient_056	9.806	0.265	2.482	113.721	103.821	141.655	0.144	14.3	6.965	0.545	0.001	0.003	15.136	0.991	0.998	0.115	10.564	0.627	0.383	0.983	107.223	98.3	98.3	98.3	0.002	3.609	0.856	0.071	6.965	21.1	0.77
patient_057	5.545	0.83	0.797	55.201	45.543	93.428	0.696	5.4	1.438	0.215	0	0.002	5.644	0.998	0.999	0.03	4.473	0.168	0.838	0.422	46.202	42.2	42.2	42.2	0.001	1.16	0.304	0.034	1.438	10.3	0.863
patient_058	9.508	0.693	1.536	127.002	109.113	172.65	0.488	12.5	2.051	0.339	0.001	0.004	14.991	0.994	0.997	0.039	10.477	0.305	0.705	0.975	109.391	97.5	97.5	97.5	0.003	2.242	0.512	0.021	2.051	27.5	0.814
patient_059	12.206	0.146	3.094	142.632	135.244	164.787	0.071	15	14.066	0.639	0.001	0.003	21.383	0.985	0.998	0.166	14.169	0.741	0.27	1.315	141.994	131.5	131.5	131.5	0.002	4.501	0.929	0.107	14.066	18.9	0.748
patient_060	6.735	0.825	0.867	92.339	71.788	149.386	0.685	7.5	1.461	0.214	0.001	0.004	8.504	0.997	0.998	0.024	6.417	0.174	0.832	0.601	71.875	60.1	60.1	60.1	0.002	1.266	0.315	0.024	1.461	23.1	0.868
patient_061	4.342	0.972	0.199	33.837	31.309	49.793	0.945	2.8	1.059	0.059	0	0.001	3.928	0.998	1	0.008	3.235	0.028	0.975	0.308	35.542	30.8	30.8	30.8	0.001	0.294	0.055	0.034	1.059	5	0.939
patient_062	5.472	0.292	2.191	57.856	49.676	92.659	0.181	4.3	5.54	0.572	0	0.002	6.405	0.994	0.999	0.175	5.005	0.581	0.427	0.471	47.307	47.1	47.1	47.1	0.001	3.177	0.819	0.118	5.54	8.8	0.706
patient_063	9.678	0.584	1.604	111.554	95.686	152.539	0.373	13.4	2.678	0.364	0.001	0.004	12.859	0.995	0.997	0.048	9.185	0.393	0.616	0.856	99.425	85.6	85.6	85.6	0.003	2.335	0.627	0.031	2.678	26.3	0.847
patient_064	3.961	0.584	1.125	51.156	37.222	105.754	0.434	3.3	2.302	0.325	0	0.002	4.164	0.997	0.999	0.067	3.409	0.344	0.661	0.324	35.704	32.4	32.4	32.4	0.001	1.631	0.566	0.071	2.302	11.5	0.849
patient_065	31.261	0.117	4.357	552.24	516.702	609.598	0.034	66.7	29.503	0.724	0.008	0.014	99.232	0.933	0.989	0.182	49.694	0.824	0.193	4.587	526.176	458.7	458.7	458.7	0.011	6.4	0.966	0.064	29.503	111.7	0.656
patient_066	1.223	0.959	0.209	14.533	12.209	30.827	0.92	1.6	1.087	0.086	0	0.001	1.322	0.998	1	0.022	1.173	0.041	0.96	0.118	12.823	11.8	11.8	11.8	0	0.304	0.08	0.093	1.087	3.5	0.873
patient_067	10.533	0.757	1.267	185.211	150.312	261.957	0.582	14.7	1.72	0.268	0.003	0.006	19.385	0.994	0.996	0.022	13.04	0.24	0.772	1.211	141.471	121.1	121.1	121.1	0.004	1.857	0.418	0.014	1.72	43.7	0.852
patient_068	12.378	0.194	2.829	186.332	164.461	235.325	0.104	17.9	9.633	0.573	0.002	0.006	24.375	0.987	0.996	0.113	15.819	0.685	0.327	1.467	162.572	146.7	146.7	146.7	0.004	4.121	0.896	0.066	9.633	38.2	0.784
patient_069	4.583	0.753	0.648	53.067	37.112	112.511	0.621	3.4	1.609	0.19	0	0.002	3.987	0.998	0.999	0.031	3.279	0.214	0.791	0.312	40.862	31.2	31.2	31.2	0.001	0.941	0.379	0.052	1.609	12.8	0.921
patient_070	11.13	0.279	2.826	131.358	123.236	155.856	0.124	12.9	8.059	0.596	0.001	0.003	19.021	0.987	0.998	0.138	12.833	0.654	0.356	1.192	125.656	119.2	119.2	119.2	0.002	4.112	0.876	0.068	8.059	18.8	0.72
patient_071	5.988	0.404	1.663	63.155	51.953	102.474	0.282	5.9	3.553	0.434	0	0.002	6.455	0.996	0.999	0.094	5.038	0.474	0.533	0.474	49.531	47.4	47.4	47.4	0.001	2.413	0.718	0.075	3.553	13.6	0.812
patient_072	11.471	0.229	2.758	163.176	140.942	215.012	0.121	15.4	8.258	0.577	0.002	0.005	19.978	0.988	0.997	0.123	13.376	0.659	0.352	1.242	132.955	124.2	124.2	124.2	0.004	4.013	0.879	0.067	8.258	35	0.751
patient_073	3.985	0.351	1.857	39.5	30.856	78.2	0.21	1.2	4.774	0.559	0	0.001	3.537	0.996	0.999	0.202	2.942	0.548	0.459	0.281	27.639	28.1	28.1	28.1	0.001	2.688	0.79	0.17	4.774	6	0.724
patient_074	15.316	0.2	3.169	226.097	202.856	275.184	0.087	22.1	11.544	0.616	0.003	0.006	31.575	0.982	0.996	0.13	19.641	0.713	0.3	1.819	199.12	181.9	181.9	181.9	0.004	4.62	0.913	0.063	11.544	44.6	0.738
patient_075	13.044	0.311	3.071	174.409	164.052	201.061	0.12	20.8	8.319	0.613	0.001	0.004	26.521	0.982	0.997	0.136	16.981	0.66	0.352	1.574	169.247	157.4	157.4	157.4	0.003	4.476	0.88	0.053	8.319	26.2	0.725
patient_076	13.167	0.336	2.861	186.593	165.051	235.068	0.14	18	7.142	0.578	0.002	0.005	24.594	0.986	0.996	0.116	15.939	0.632	0.38	1.478	160.654	147.8	147.8	147.8	0.004	4.167	0.86	0.048	7.142	37.8	0.738
patient_077	12.093	0.46	2.344	167.736	148.555	211.009	0.246	20.2	4.069	0.484	0.002	0.005	21.716	0.989	0.996	0.073	14.353	0.509	0.502	1.332	147.723	133.2	133.2	133.2	0.004	3.417	0.754	0.031	4.069	37	0.759
patient_078	13.774	0.276	3.004	217.508	192.366	272.634	0.11	19.1	9.12	0.591	0.002	0.006	29.329	0.983	0.996	0.117	18.468	0.676	0.337	1.711	186.279	171.1	171.1	171.1	0.004	4.381	0.89	0.053	9.12	40.1	0.745
patient_079	14.008	0.206	3.221	165.954	157.416	189.872	0.088	16.9	11.413	0.647	0.001	0.004	25.553	0.981	0.998	0.164	16.46	0.711	0.301	1.526	161.042	152.6	152.6	152.6	0.002	4.692	0.912	0.075	11.413	21.7	0.682
